# The Super-scan and Flare Phenomena in a Nasopharyngeal Cancer Patient: A Case Report

**DOI:** 10.4021/jocmr820w

**Published:** 2012-05-15

**Authors:** Min-Chuan Lu, Tzyy-Ling Chuang, Moon-Sing Lee, Wen-Yen Chiou, Hon-Yi Lin, Shih-Kai Hung

**Affiliations:** a Department of Radiation Oncology, Buddhist Dalin Tzu Chi General Hospital, Chiayi, Taiwan; b Department of Nuclear Medicine, Buddhist Dalin Tzu Chi General Hospital, Chiayi, Taiwan; c School of Medicine, Tzu Chi University, Hualien, Taiwan

**Keywords:** Flare phenomenon, Nasopharyngeal cancer, Superscan

## Abstract

A 26-year-old man with a history of nasopharyngeal carcinoma (NPC) presented with bone metastasis. Bone scan revealed diffuse skeletal metastases with superscan appearance. Afterward, radiotherapy for bone lesions was arranged and the effects were evaluated. The bone scan flare phenomenon appeared within a short time after radiotherapy. Diffuse bone metastasis, which is common in NPC, were indeterminate on images showing superscan pattern or flare.

## Introduction

Nasopharyngeal carcinoma (NPC) is a unique malignant head and neck cancer with a specific behavior. It is rarely reported in the West but occurs at high frequency in Southern China, Hong Kong, Taiwan, Singapore, and Malaysia [1]. Radiotherapy has long been the standard treatment for patients with NPC because of its anatomic location and relative radiosensitivity. However, a high rate of distant failure is observed in patients with advanced NPC. NPC tends to metastasize to bone, lung, and liver [2]. Bone imaging using technetium-99m methylene diphosphonate (Tc-99m MDP) is one of the most frequently used nuclear imaging techniques to examine bone lesions [3]. However, diffuse metastasis, commonly seen in prostate cancer or breast cancer, are indeterminate on images showing superscan and flare phenomena [4]. We reported a case of NPC manifesting superscan and flare phenomena on bone images.

## Case Report

**Figure 1:**
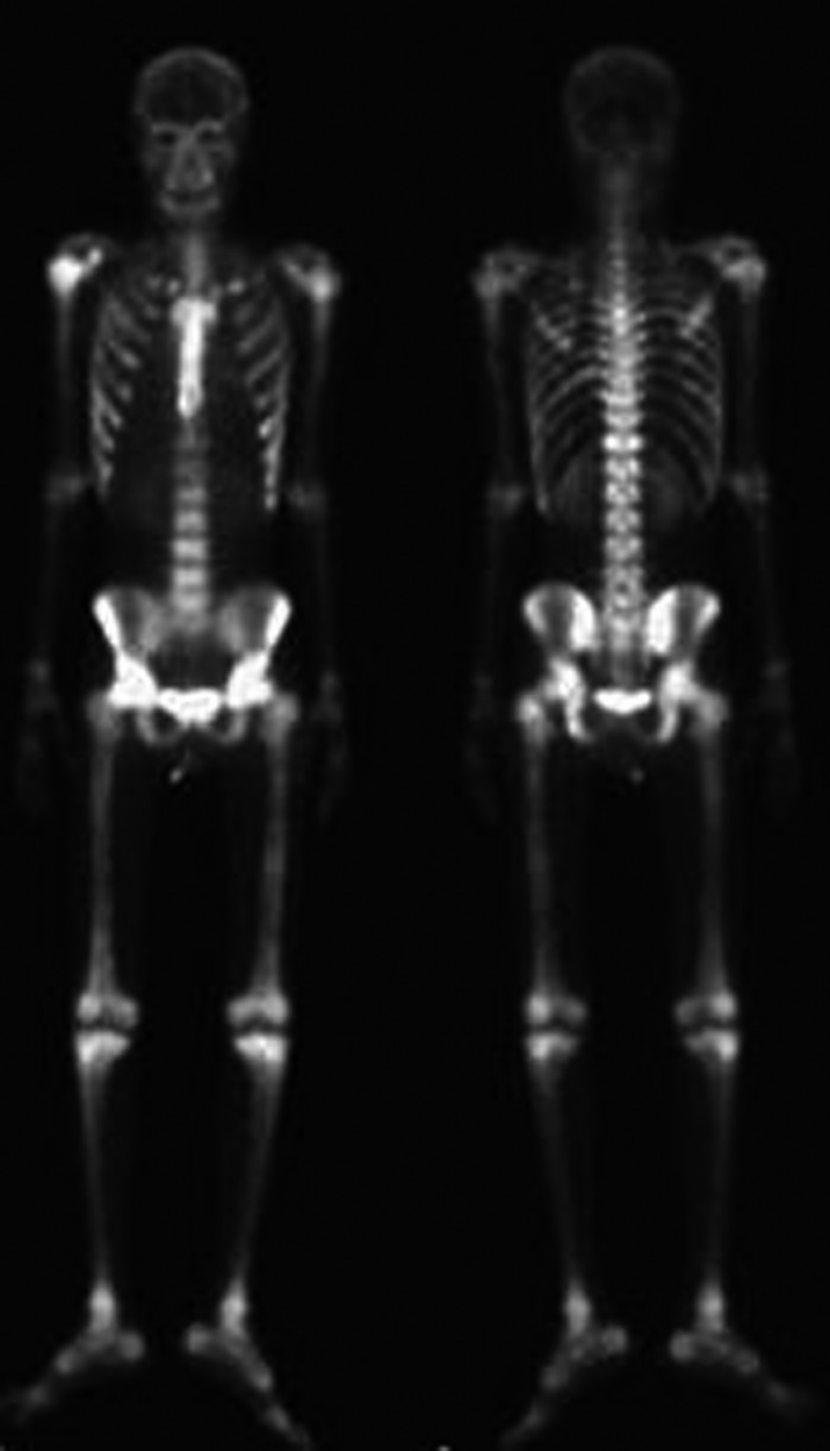
Bone scan image reveals diffuse and increased uptake before radiotherapy.

**Figure 2:**
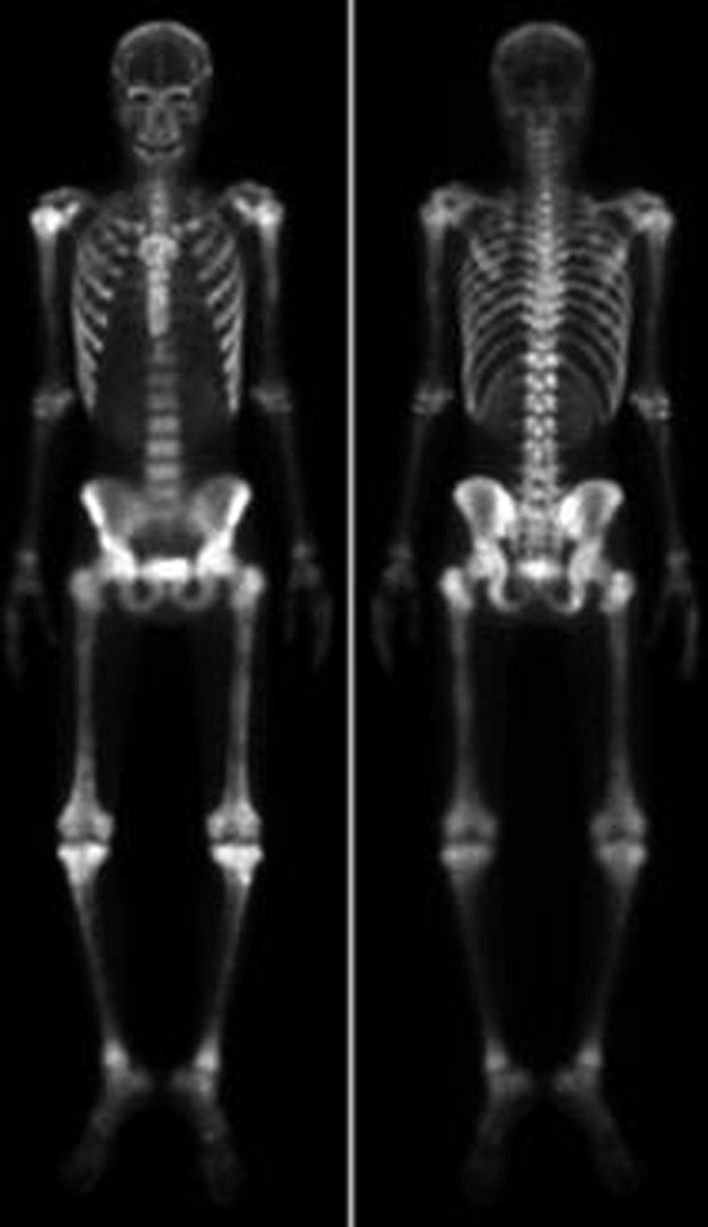
Bone scan image reveals diffuse and increased uptake 3 months after radiotherapy.

**Figure 3:**
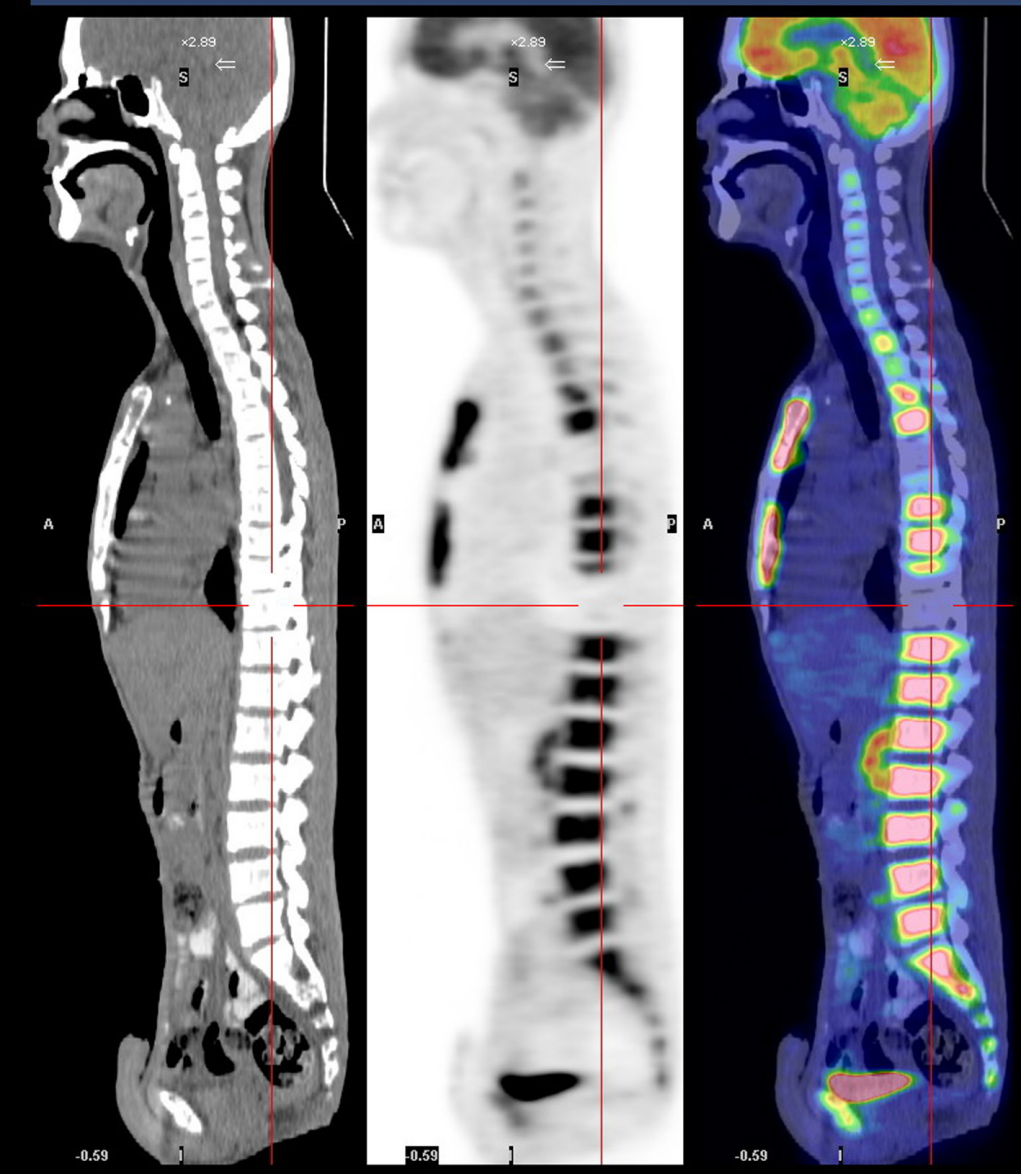
PET scan image reveals “cold” lesions in areas which received radiotherapy.

A 26-year-old man was diagnosed with malignant NPC, which, after treatment, metastasized to bone. At the beginning, he received full cycles of chemotherapy and radiotherapy. At his regular follow up, back pain was noted after definitive treatment for 3 months. So we further investigated the progression of his disease by bone scanning. The bone scan revealed diffuse increased tracer uptake throughout the skeleton (Fig. 1). Because diffuse metastases were highly suspected and superscan pattern was revealed, we also arranged MRI of the spine to confirm the presence of bone lesions. Diffuse bony metastases were detected. With disease progression, back pain was exacerbated and poorly controlled by pain killer medications. Localized radiation treatment (300 cGy in 10 fractions) to vertebrae T5, T6, T10, and the lower portion of T9, and 3 months after radiotherapy, a bone scan to detect the effects, were arranged. However, increased diffuse activity indicating diffuse metastases was still present (Fig. 2). Examination by 18-fluoro-2-deoxyglucose positron emission tomography (FDG-PET) to determine the extent of metastatic spread demonstrated multiple bony metastatic lesions and enhanced uptake in the skull, entire spine, rib cage, pelvic bones, and humeral and femoral shafts. Additionally, the PET scan also revealed “cold” lesions in areas which had received radiotherapy (Fig. 3).

## Discussion

Bone scan is a very sensitive method to screen for bone abnormalities, especially in cancer patients. About 75% of patients with malignancy and pain have abnormal bone scintigraphic findings [5]. A super scan pattern is characterized by a strikingly high bone-to-soft-tissue ratio on skeletal scintigraphy. When the bone is hyper-metabolic, the absorption of isotope by the bone relative to soft tissue and kidneys will become greater, and signal from the kidneys will consequently be lost. The super scan pattern can be seen in a variety of malignant diseases, including breast, lung, and prostate cancers [6]. Figure 1 shows the super scan pattern in a patient with NPC.

To evaluate the effect of the treatment, we arranged another bone scan to monitor the bone lesions after radiotherapy. Interestingly, a flare phenomenon became apparent within short time after radiotherapy. A flare response on Tc-99m bone scan indicates progression of bone metastasis despite the effectiveness of treatment. Inflammation might increase blood flow or the healing reaction might increase bone turnover [7, 8]. However, the question remains of why a flare reaction is not seen more often. Pollen et al. reported flare reaction in 6% of prostate cancer patients 3 months after initiation of treatment [4]. In our case, a PET scan was arranged to make a differential diagnosis. The PET scan revealed that the lesions in areas which had received radiotherapy were “cold” (Fig. 3). But the Tc-99m MDP bone scan showed a uniform symmetrical increase in bone uptake of tracer and diminished to absent renal uptake of tracer. Moreover, those areas of the spine that received radiotherapy showed a flare response (Fig. 2). It seems that the flare reaction briefly affects the bone scan by reducing its capacity to reveal bone lesions that had received radiotherapy.

Thus, the results of bone scans can sometimes be obscured by phenomena such as super scan pattern and flare reaction. PET scan can reveal the real bone lesions.
